# Trends in and correlates of unhealthy alcohol consumption among United States Veterans, 2016–2024

**DOI:** 10.1016/j.abrep.2026.100717

**Published:** 2026-06-06

**Authors:** Vuong V. Do, Weisiyu Abraham Qin, Mark R. Hawes, Salomeh Keyhani, Katherine J. Hoggatt, Pamela M. Ling

**Affiliations:** a Center for Tobacco Control Research and Education, University of California, San Francisco, San Francisco, CA, USA; b Division of General Internal Medicine, Department of Medicine, University of California, San Francisco, San Francisco, CA, USA; c Center for Data to Discovery and Delivery Innovation (3DI), San Francisco Veterans Affairs Health Care System, San Francisco, CA, USA

**Keywords:** Veteran, Alcohol, Binge drinking, Heavy drinking, Trend, BRFSS, Military

## Abstract

**Background:**

Unhealthy alcohol consumption remains a significant public health concern among U.S. Veterans, yet less is known about recent trends in binge and heavy drinking among this population. This study examines trends in and correlates of binge and heavy alcohol use among Veterans across age and sex.

**Methods:**

This study used nationally representative Behavioral Risk Factor Surveillance System (BRFSS) data from 2016 to 2024. Average annual percent change (AAPC) was estimated using join-point regression. Multivariable logistic regression was used to examine correlates of binge and heavy drinking.

**Results:**

From 2016 to 2024, binge drinking declined modestly overall (15.8% to 14.6%, AAPC = −1.2%, *p* < .05), with significant decreases among Veterans aged 18–34 and 65–80 (−2.1% and − 2.6%, *p* < .05, respectively). Heavy drinking remained stable overall but increased among Veterans age 35–49 (7.4% to 9.6%, AAPC = 2.7%, *p* < .05). Men consistently demonstrated higher binge drinking rates. Women exhibited a notable decline in heavy drinking (8.5% to 6.3%, AAPC = -2.8%, *p* < .05), converging with men by 2024. Veterans with military-related health insurance were less likely to engage in binge drinking, while those reporting cost-related barriers to care and current tobacco use had higher odds of heavy drinking. A graded relationship was observed between poor mental health and heavy drinking.

**Conclusions:**

Despite overall declines, binge and heavy drinking remain prevalent among Veterans, with emerging midlife risks and sex-specific trajectories. Age- and sex-specific prevention, integrated tobacco and alcohol treatment, and continued efforts to reduce financial barriers to care may reduce alcohol use.

## Highlights

• Binge drinking among U.S. Veterans declined from 2016 to 2024, especially among those aged 18–34 and 65–80.
• Heavy drinking remained stable overall but increased significantly among midlife Veterans aged 35–49.
• Sex differences narrowed over time, as female veterans' heavy drinking declined.
• Military-related insurance was linked to lower binge drinking, while tobacco use and care barriers were linked to heavy drinking.
• Poor mental health showed a graded association with heavy drinking.

## 1 Introduction

Alcohol contributes to adverse health outcomes among U.S. Veterans (Hoggatt et al., 2021), with unhealthy use (i.e., binge and heavy drinking) linked to morbidity, premature mortality, and reduced quality of life (Chwastiak et al., 2010; Oppezzo et al., 2016). Veterans consistently report higher rates of alcohol use than the general population (Carr et al., 2021), driven by combat-related trauma, posttraumatic stress disorder (PTSD), transition stress, and social isolation (Kelley et al., 2013). In addition, military and veteran cultural norms may normalize and reinforce alcohol use as part of social bonding, decompression, camaraderie, and group belonging (Meadows et al., 2023).

Binge drinking and heavy drinking are distinct alcohol-use patterns. Past 30-day binge drinking (4 or more drinks for women or 5 or more drinks for men on a single occasion) captures episodic high-intensity drinking, whereas, heavy drinking (more than 7 drinks per week for women or more than 14 drinks per week for men) reflects sustained exposure linked to chronic health risks (Centers for Disease Control and Prevention, 2025a). Examining both patterns provides a more comprehensive understanding of alcohol-related risk among Veterans.

In 2023, 42% of Veterans who consumed alcohol reported past-30 day binge drinking, and nearly 16% met criteria for heavy drinking, exceeding general population trends (Davis et al., 2025). Few studies capture differences by age or sex or include associations with social risk factors. Risky drinking varies by age, with highest levels among younger Veterans (Wong et al., 2024). Among female Veterans, excessive alcohol use may carry disproportionate harms, including greater vulnerability to alcohol-related health consequences and trauma-related coping behaviors (Rodriguez et al., 2024). These risks may be further shaped by exposure to military sexual trauma or evolving social norms around alcohol use (Gil et al., 2024). The female veteran population is expected to exceed 2 million by 2025 (Gil et al., 2024), but few studies address trends in unhealthy alcohol consumption among female Veterans. Thus, understanding how unhealthy alcohol use varies by age and sex is critical for identifying high-risk subgroups to inform targeted prevention and intervention strategies.

To fill these gaps, this study examined: 1) the trends in binge and heavy drinking among Veterans stratified by age and sex, and 2) factors associated with binge and heavy drinking.

## 2 Methods

### 2.1 Data and participants

This cross-sectional study used 2016–2024 Behavioral Risk Factor Surveillance System (BRFSS), a nationally representative telephone survey of U.S. adults. The BRFSS is conducted annually across all 50 states and participating U.S. territories to collect information on health-related behaviors. This study combined multiple survey years of repeated annual cross-sectional samples, and participants were not followed longitudinally. Observed trends therefore reflect population-level changes rather than within-person changes overtime. During COVID-19 pandemic, BRFSS continued to collect data using standardized landline and cellular telephone interviews; however, pandemic-related disruptions may have affected survey operations, participation, and response patterns. BRFSS employs complex sampling weights to account for unequal selection probabilities, nonresponse, and population representation; these weights were applied in all analyses. Survey design details are described elsewhere (Centers for Disease Control and Prevention, 2024). This study used publicly available, deidentified data and, thus, was exempt from institutional review board review.

Participants' veteran status was identified as respondents who answered “yes” to the question: *“Have you ever served on active duty in the United States Armed Forces, either in the regular military or in a National Guard or military reserve unit?”* Only Veterans with non-missing responses for past 30-day alcohol use were included, resulting in an analytic sample of 455,129 Veterans over 9 survey years (details by year in Supplemental Table 1).

### 2.2 Measures

Outcome variables were past 30-day binge drinking and heavy drinking. Covariates included age (18–34, 35–49, 50–64, 65–80), self-reported sex (male, female), marital status (married; divorced/widowed/separated; never married; a member of an unmarried couple), educational attainment (less than high school/high school graduate/some college or technical school/college graduate), and race/ethnicity (Hispanic, non-Hispanic White, non-Hispanic Black, non-Hispanic multiracial, and non-Hispanic other). Other covariates of unhealthy alcohol use were selected based on previous studies (Wilson et al., 2018), such as general health status (poor, fair, good, very good/excellent), primary source of health insurance coverage (military-related health insurance, other type of insurance, no coverage of any type), and inability to obtain needed medical care due to cost within the past 12 months (yes/no). Mental health status was assessed using self-reported number of poor mental health days in the past 30 days, categorized as 0, 1–5, 6–10, or 11–30. Current tobacco use (yes/no) was based on self-reported use of cigarettes, smokeless tobacco, or e-cigarettes either daily or some days.

### 2.3 Statistical analysis

For aim 1, weighted prevalence of binge drinking and heavy drinking were estimated for each age group and sex. In addition, average annual percent changes (AAPCs) were estimated for each outcome variable, using log-linear regression models to assess temporal patterns and quantify the direction and magnitude of changes in prevalence (Clegg et al., 2009). For aim 2, we combined data from the 2023 and 2024 surveys (two years after the COVID-19 pandemic) to examine factors associated with binge and heavy drinking among Veterans, using multivariable binary logistic regression. 2023 and 2024 data were combined to increase the power of the sample and to focus on the post-acute pandemic period, acknowledging that residual pandemic-related influences may persist. Final sample weights were adjusted following CDC guidelines (Centers for Disease Control and Prevention, 2025b), accounting for the proportion of the sample size contributed by each state relative to the total annual sample for each survey year. All covariates were included simultaneously in multivariable logistic regression models for both binge and heavy drinking outcomes. Statistical significance was determined using 2-sided tests with alpha = 0.05. Data cleaning and analyses were conducted using Stata version 18 (StataCorp LLC), and AAPCs were estimated using the Joinpoint Regression Program version 5.4.0.0, National Cancer Institute.

## 3 Results

### 3.1 Trends in binge and heavy drinking

**Fig. 1:**
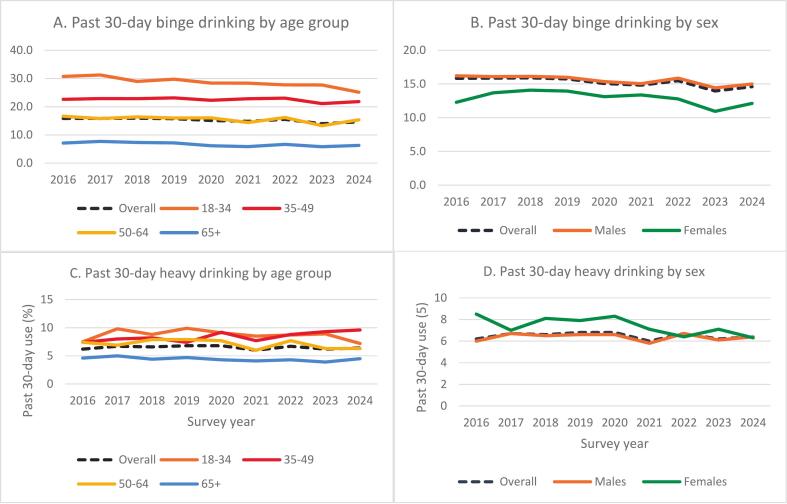
Past 30-day binge drinking and heavy drinking prevalence among Veterans, stratified by age group and sex.

Trends in binge and heavy drinking and AAPCs are presented in Fig. 1 and Supplementary Table 1. Overall, binge drinking among Veterans declined slightly from 15.8% (95% confidence intervals [CI]: 15.2%, 16.5%) in 2016 to 14.6% (95% CI: 13.8%, 15.4%) in 2024, corresponding to an AAPC of −1.2% (95% CI: −2.2%, −0.3%) **(**Fig. 1A**)**. The decline was significant among Veterans aged 18–34 years (from 30.7% to 25.1%; AAPC = −2.1%, 95% CI: −3.0%, −1.3%) and those aged 65–80 years (from 7.1% to 6.3%; AAPC = −2.6%, 95% CI: −4.1%, −1.0%). Prevalence remained stable among Veterans aged 35–49 and 50–64 years, with the prevalence being 21.8% (95% CI: 19.6%, 24.2%) and 15.3% (95% CI: 14.0%, 16.7%) in 2024, respectively. In addition, binge drinking was consistently lower among female Veterans than males and showed no significant trend over time in either group (Fig. 1B). In 2024, prevalence was 12.1% (95% CI: 10.2%, 14.4%) among females versus 15% (95%CI: 14.2%, 15.8%) among male Veterans.

Heavy drinking remained stable, with prevalence 6.4% (95% CI: 5.8%, 6.9%) in 2024 (Fig. 1C). No significant changes were observed across most age groups, except for those aged 35–49 years, where prevalence increased from 7.4% (95% CI: 6.3%, 8.8%) in 2016 to 9.6% (95% CI: 7.7%, 11.9%) in 2024 (AAPC = 2.7%, 95% CI: 0.7%, 4.7%). Heavy drinking prevalence was higher among female Veterans than males in some years during 2016–2021 but declined significantly thereafter (Fig. 1D, AAPC = −2.8%, 95% CI: −4.6%, −0.8%), reaching comparable levels in 2024 (male Veterans: 6.4%, 95% CI: 5.8%, 7.0% vs. female Veterans: 6.3%, 95% CI: 5.2%, 7.5%).

### 3.2 Associated factors of binge and heavy drinking

Factors associated with binge and heavy drinking are shown in Table 1. Younger Veterans had higher odds of binge and heavy drinking compared to those of the 65–80 years. Male Veterans had higher odds of binge drinking (adjusted odds ratio [AOR] = 1.81, 95% CI: 1.56, 2.10) compared to female Veterans, but the odds of heavy drinking were not significantly different between males and females. Veterans identifying as non-Hispanic Black or non-Hispanic Other were less likely to report binge or heavy drinking than non-Hispanic White Veterans. Those with military-related health insurance had lower odds of binge drinking (AOR = 0.86, 95% CI: 0.77, 0.95) compared to those with other types of insurance. Veterans who could not see a doctor due to cost were more likely to report heavy drinking (AOR = 1.44, 95% CI: 1.01, 2.07).

**Table 1:** Factors associated with binge and heavy drinking among Veterans, 2023 & 2024 behavioral risk factor surveillance system.

Covariates	Binge drinking	Heavy drinking
	Adjusted ORs (95% CI)	Adjusted ORs (95% CI)
Age		
*65–80 (Reference)*	1.00	1.00
*18–34*	4.23 (3.61, 4.95) ***	1.26 (1.02, 1.57) *
*35–49*	3.45 (2.99, 3.97) ***	1.72 (1.40, 2.10) ***
*50–64*	2.28 (2.02, 2.57) ***	1.23 (1.06, 1.43) ***
Sex (Male vs. Female)	1.81 (1.56, 2.10) ***	0.99 (0.82, 1.20)

Race/ethnicity		
*White, non-Hispanic (Reference)*	1.00	1.00
*Black, non-Hispanic*	0.69 (0.59, 0.82) ***	0.54 (0.43, 0.67) ***
*Hispanic*	1.06 (0.89, 1.27)	1.02 (0.71, 1.44)
*Multiracial, non-Hispanic*	0.94 (0.74, 1.19)	0.89 (0.63, 1.25)
*Other, non-Hispanic*	0.70 (0.55, 0.89) **	0.59 (0.41, 0.83) **

Marital status		
*Married (Reference)*	1.00	1.00
*Divorced/widowed/separated*	1.11 (1.01, 1.25) *	1.12 (0.96, 1.31)
*Never married*	1.13 (0.98, 1.31)	0.96 (0.75, 1.23)
*A member of an unmarried couple*	1.13 (0.91, 1.40)	0.93 (0.64, 1.36)

Education level		
*Less than high school (Reference)*	1.00	1.00
*Graduated from high school*	0.89 (0.67, 1.19)	0.82 (0.56, 1.21)
*Attended colleges or technical schools*	0.89 (0.67, 1.18)	0.79 (0.54, 1.14)
*Graduated from colleges or technical schools*	0.80 (0.60, 1.07)	0.71 (0.49, 1.03)

Primary Source of Health Insurance		
*Other sources*	1.00	1.00
*Military related health care*	0.86 (0.77, 0.95) **	0.99 (0.84, 1.16)
*No coverage of any type*	0.82 (0.63, 1.07)	1.14 (0.79, 1.68)
Could not see doctors because of the cost (yes vs. no)	1.11 (0.88, 1.39)	1.44 (1.01, 2.07) *

Self-reported general health status		
*Very good/excellent (Reference)*	1.00	1.00
*Good*	0.98 (0.88, 1.09)	0.89 (0.75, 1.05)
*Fair*	0.76 (0.65, 0.90) **	0.69 (0.55, 0.87) **
*Poor*	0.52 (0.42, 0.65) ***	0.76 (0.56, 1.04)
Current tobacco use (yes vs. no)	2.26 (2.04, 2.51) ***	2.61 (2.25, 3.04) ***

Number of days mental health was not good in the past 30 days		
*0 day (Reference)*	1.00	1.00
*1–5 days*	1.42 (1.24, 1.64) ***	1.26 (0.99, 1.60)
*6–10 days*	1.52 (1.26, 1.83) ***	1.60 (1.21, 2.13) **
*11–30 days*	1.47 (1.28, 1.70) ***	1.75 (1.42, 2.15) ***

Notes: OR: Odds ratio, CI: Confidence Interval, ^⁎⁎⁎^*P* < .001, ^⁎⁎^*P* < .01, ^⁎^*P* < .05.

Unweighted sample *n* = 87,762 Veterans (weighted population size = 21,526,942).

Binge drinking was defined as 5 or more drinks for adult males or 4 or more drinks for adult females on an occasion in the past 30 days. Heavy drinking was defined as more than 14 drinks per week for adult males or more than 7 drinks per week for adult females.

Veterans currently using tobacco had more than twice the odds of binge (AOR = 2.26, 95% CI: 2.04, 2.51) and heavy drinking (AOR = 2.61, 95% CI: 2.25, 3.04). Poor mental health demonstrated a dose-response relationship with heavy drinking.

## 4 Discussion

Overall, binge drinking among Veterans declined modestly from 2016 to 2024, with the largest decreases among the youngest (18–34) and oldest (65–80) groups. In contrast, heavy drinking remained stable overall but rose among Veterans ages 35–49. This divergence may reflect generational and life-stage dynamics among Veterans. Midlife is often marked by occupational and caregiving strain in veteran populations, which may contribute to increases in heavy drinking (Osborne et al., 2022).

Sex differences in alcohol use among Veterans have shown inconsistent patterns in previous research (Fear et al., 2007). The current study found males consistently exhibited higher levels of binge drinking compared to females and the trends remained stable in both sexes during the period. The trend in heavy drinking among females exhibited a notable decline over time and reached the same level as males in 2024, aligning with reports that sex gaps could narrow over time in veteran samples (Hoggatt et al., 2015; Osborne et al., 2022). This pattern may be consistent with broader evidence of narrowing sex differences in alcohol-related risk (Shuey et al., 2025), or may reflect veteran-specific changes in care access and service engagement. Over the past two decades, the VA health care system has expanded its focus on women Veterans' health, including comprehensive women's health services, mental health care, and substance use-related resources (Borsky et al., 2025), which may have improved opportunities for screening, early identification, counseling, and linkage to treatment.

Veterans with military-related primary health insurance coverage had lower odds of binge drinking, consistent with Veterans Health Administration (VA) policies that promote routine alcohol screening and access to care (Chen et al., 2018). These integrated models may help mitigate risky drinking patterns. On the other hand, Veterans facing financial constraints are more likely to delay or ignore care, including prevention and treatment services, which might have contributed to the persistence or escalation of heavy drinking observed in this study.

The robust associations between unhealthy alcohol use and tobacco use, and mental health burden were consistent with prior research documenting clustering of behavioral health risks among Veterans (Cooper et al., 2019). These relationships may reflect shared vulnerability, including common neurobiological pathways, co-occurring psychosocial stressors, and the use of substances as coping or self-medication mechanisms (Hua et al., 2023; Osborne et al., 2022).

Study findings support maintenance and expansion of universal alcohol screening and brief intervention in primary care for Veterans, alongside low-threshold referrals. The observed increases among midlife Veterans suggest that this group may warrant additional monitoring and outreach, although future research is needed to determine the most effective screening, intervention, and service-delivery approaches. Given the co-occurrence of tobacco use, mental health burden, and unhealthy alcohol use, integrated assessment of these behavioral health risks may be useful in veteran-serving care settings. Finally, reducing cost-related barriers to non-VA or community-based services may help lower the risk of heavy drinking among Veterans.

This study has several limitations. First, self-reported measures, nonresponse bias, and unmeasured factors such as genetic predisposition or deployment history, may influence findings. Some fluctuations around 2020 may reflect pandemic-related changes in alcohol use, although this study was not designed to isolate these effects. Finally, operational definitions of binge and heavy drinking in BRFSS may not fully capture all drinking behavior. Future studies should evaluate military sexual trauma and deployment history, which were not available in the present analysis but may influence unhealthy alcohol use among Veterans.

## 5 Conclusions

This large, nationally representative study quantified age- and sex-stratified trends in alcohol use and binge and heavy drinking among Veterans. Sustained surveillance efforts particularly for midlife Veterans, integrating tobacco and alcohol treatment, expanding sex-responsive and trauma-informed services, and addressing social determinants of alcohol use may help reduce risk. Future research should prioritize longitudinal designs that include Veterans within and outside the VA healthcare system, and linking survey and clinical data to establish temporal relationships and explore potential mechanisms underlying alcohol use behavior.

## Author’s contribution

VVD conceptualized the study, conducted the analysis, and contributed to drafting the initial manuscript and to all subsequent drafts. WAQ conceptualized the study and contributed to drafting the initial manuscript and to all subsequent drafts. MRH, SK, KJH contributed to interpretation of findings and reviewed and revised the manuscript. PML conceptualized the study, contributed to analyses and interpretation of findings, and reviewed and revised the manuscript. All authors have read and approved the final manuscript for submission.

## CRediT authorship contribution statement

**Vuong V. Do:** Writing – review & editing, Writing – original draft, Formal analysis, Conceptualization. **Weisiyu Abraham Qin:** Writing – review & editing, Writing – original draft, Conceptualization. **Mark R. Hawes:** Writing – review & editing. **Salomeh Keyhani:** Writing – review & editing. **Katherine J. Hoggatt:** Writing – review & editing. **Pamela M. Ling:** Writing – review & editing, Conceptualization.

## Declaration of competing interest

The authors declare that they have no known competing financial interests or personal relationships that could have appeared to influence the work reported in this paper.

## Data Availability

Data will be made available on request.

## Appendix A Supplementary data

Supplementary Table 1. Prevalence of binge and heavy drinking among veterans by age group and sex from 2016 to 2024
