# Supplementary material for: Trends in and correlates of unhealthy alcohol consumption among United States Veterans, 2016–2024
Source: Addict Behav Rep. 2026 Jun 6;24:100717. doi: 10.1016/j.abrep.2026.100717 (PMC13276145; doi:10.1016/j.abrep.2026.100717)
Supplement: Supplementary file 1 — Supplementary Table 1. Prevalence of binge and heavy drinking among veterans by age group and sex from 2016 to 2024 [file mmc1.docx]

**Supplementary Table 1.** Prevalence of binge and heavy drinking among veterans by age group and sex from 2016 to 2024

|  | **2016** | **2017** | **2018** | **2019** | **2020** | **2021** | **2022** | **2023** | **2024** | **AAPC, %** |
| --- | --- | --- | --- | --- | --- | --- | --- | --- | --- | --- |
|  | **n=60,190** | **n=54,205** | **n=52,923** | **n=50,191** | **n=45,131** | **n=48,899** | **n=48,214** | **n=47,653** | **n=47,723** |  |
|  | **N=25,209,228** | **N=24,515,708** | **N=25,630,687** | **N=23,964,460** | **N=24,233,551** | **N=22,165,745** | **N=23,168,403** | **N=22,727,323** | **N=22,613,674** |  |
| **Binge drinking** | | | | | | | | | | |
| Overall | 15.8  (15.2, 16.5) | 15.9  (15.2, 16.6) | 15.9  (15.2, 16.6) | 15.7  (15.1, 16.4) | 15.1  (14.3, 15.9) | 14.8  (14.1, 15.6) | 15.5  (14.8, 16.2) | 14.0  (13.2, 14.8) | 14.6  (13.8, 15.4) | -1.2  (-2.2, -0.3)* |
| *18-34* | 30.7  (28.4, 33.1) | 31.2  (28.8, 33.7) | 28.9  (26.6, 31.4) | 29.7  (27.2, 32.3) | 28.4  (25.6, 31.3) | 28.3  (25.9, 30.9) | 27.7  (25.4, 30.2) | 27.7  (24.7, 31.0) | 25.1  (22.5, 27.9) | -2.1  (-3.0, -1.3)* |
| *35-49* | 22.6  (20.8, 24.5) | 22.9  (20.9, 25.0) | 22.8  (20.9, 24.9) | 23.1  (21.2, 25.1) | 22.3  (19.8, 24.9) | 22.8  (20.6, 25.2) | 23.0  (21.2, 24.9) | 21.1  (18.9, 23.4) | 21.8  (19.6, 24.2) | -0.5  (-1.5, 0.4) |
| *50-64* | 16.6  (15.4, 17.9) | 15.8  (14.4, 17.2) | 16.4  (14.8, 18.1) | 16.0  (14.7, 17.3) | 16.0  (14.6, 17.6) | 14.4  (13.2, 15.6) | 16.2  (14.8, 17.6) | 13.3  (12.1, 14.5) | 15.3  (14.0, 16.7) | -1.6  (-3.6, 0.4) |
| *65-80* | 7.1 (6.5, 7.7) | 7.7 (7.0, 8.4) | 7.3 (6.6, 8.0) | 7.2 (6.6, 7.8) | 6.1 (5.5, 6.9) | 5.8 (5.2, 6.5) | 6.6 (6.1, 7.2) | 5.8 (5.2, 6.5) | 6.3 (5.7, 6.8) | -2.6  (-4.1, -1.0)* |
| *Males* | 16.2  (15.6, 16.9) | 16.1  (15.4, 16.8) | 16.1  (15.4, 16.9) | 16.0  (15.3, 16.7) | 15.3  (14.5, 16.2) | 15.0  (14.3, 15.8) | 15.9  (15.1, 16.6) | 14.4  (13.5, 15.3) | 15.0  (14.2, 15.8) | -1.1  (-2.0, -0.3)* |
| *Females* | 12.3  (10.4, 14.5) | 13.7  (11.9, 15.7) | 14.1  (12.0, 16.4) | 13.9  (11.7, 16.6) | 13.1  (10.8, 15.8) | 13.4  (11.4, 15.6) | 12.8  (11.0, 14.8) | 10.9  (9.3, 12.8) | 12.1  (10.2, 14.4) | -1.0  (-3.1, 0.6) |
| **Heavy drinking** | | | | | | | | | | |
| Overall | 6.2 (5.8, 6.7) | 6.7 (6.3, 7.2) | 6.6 (6.2, 7.1) | 6.8 (6.3, 7.3) | 6.8 (6.3, 7.4) | 6.0 (5.5, 6.5) | 6.7 (6.2, 7.2) | 6.2 (5.6, 6.9) | 6.4 (5.8, 6.9) | -0.2  (-2.0, 1.4) |
| *18-34* | 7.5 (6.3, 9.1) | 9.8  (8.4, 11.4) | 8.8  (7.4, 10.4) | 9.9  (8.3, 11.7) | 9.1  (7.5, 11.2) | 8.5  (7.0, 10.3) | 8.7  (7.2, 10.5) | 8.9  (6.4, 12.1) | 7.2 (5.8, 9.0) | -1.1  (-4.8, 1.7) |
| *35-49* | 7.4 (6.3, 8.8) | 8.0 (6.7, 9.6) | 8.2 (7.0, 9.5) | 7.4 (6.3, 8.6) | 9.2  (7.5, 11.2) | 7.7 (6.6, 8.9) | 8.8  (7.5, 10.2) | 9.3  (7.7, 11.0) | 9.6  (7.7, 11.9) | 2.7  (0.7, 4.7)* |
| *50-64* | 7.4 (6.5, 8.3) | 6.9 (6.1, 7.8) | 7.9 (6.8, 9.1) | 7.9 (7.0, 9.0) | 7.7 (6.7, 8.8) | 6.0 (5.3, 6.8) | 7.7 (6.8, 8.7) | 6.3 (5.5, 7.2) | 6.3 (5.6, 7.2) | -1.9  (-7.5, 4.2) |
| *65-80* | 4.6 (4.2, 5.1) | 5.0 (4.4, 5.6) | 4.4 (4.0, 4.9) | 4.7 (4.3, 5.2) | 4.3 (3.8, 4.8) | 4.1 (3.4, 5.0) | 4.3 (3.8, 4.9) | 3.9 (3.4, 4.4) | 4.5 (4.0, 5.0) | -1.4  (-3.2, 0.2) |
| *Males* | 6.0 (5.6, 6.4) | 6.7 (6.2, 7.2) | 6.5 (6.0, 7.0) | 6.6 (6.2, 7.1) | 6.6 (6.0, 7.1) | 5.8 (5.3, 6.4) | 6.7 (6.2, 7.3) | 6.1 (5.4, 6.9) | 6.4 (5.8, 7.0) | 0.2  (-1.5, 1.6) |
| *Females* | 8.5  (6.7, 10.7) | 7.0 (5.8, 8.5) | 8.1 (6.6, 9.8) | 7.9  (6.2, 10.0) | 8.3  (6.5, 10.6) | 7.1 (5.7, 8.7) | 6.4 (5.4, 7.7) | 7.1 (5.9, 8.6) | 6.3 (5.2, 7.5) | -2.8  (-4.6, -0.8)* |

*Notes:* n = unweighted sample size; N = corresponding weighted veteran population; AAPC = average annual percent change; * AAPC was statistically significantly different from 0. Values represent prevalence estimates for each survey year, with 95% confidence intervals shown in parentheses.
